# Supplementary material for: A somatic evolutionary model of the dynamics of aneuploid cells during hematopoietic reconstitution
Source: Sci Rep. 2020 Jul 22;10:12198. doi: 10.1038/s41598-020-68729-1 (PMC7376010; doi:10.1038/s41598-020-68729-1)
Supplement: Supplementary file 1 — Supplementary information. [file 41598_2020_68729_MOESM1_ESM.pdf]

SUPPLEMENTARY INFORMATION

A somatic evolutionary model of the dynamics of aneuploid cells during hematopoietic reconstitution

Andrii I. Rozhok[1,], Rebecca E. Silberman[2,3,4], Kelly C. Higa[1,5], L. Alex Liggett[1], Angelika Amon[2,3,4], James DeGregori[1,5,6,7,*]*

[1]Department of Biochemistry and Molecular Biology, University of Colorado School of Medicine, Aurora, CO 80045

[2]David H. Koch Institute for Integrative Cancer Research, Massachusetts Institute of Technology, Cambridge, Massachusetts 02139, USA;

[3]Howard Hughes Medical Institute, Massachusetts Institute of Technology, Cambridge, Massachusetts 02139, USA;

[4]Department of Biology, Massachusetts Institute of Technology, Cambridge, Massachusetts 02139, USA

[5]Integrated Department of Immunology, [6]Department of Pediatrics, [7]Department of Medicine, Section of Hematology, University of Colorado School of Medicine, Aurora, CO 80045

*Correspondence to andrii.rozhok@cuanschutz.edu and james.degregori@cuanschutz.edu

Model code

```
hold off

% 1. Change 2nd var in clearvars to the titrated
% 2. Change a)nConds + b)the 1st var in the c-loop to the titrated
% 3. Check all the vars just below

clearvars legs

cellDivRateRange = [3, 10, 20, 40, 60];
initPopSizeRange = [1000 5000 10000 25000 50000];
maxPopSizeRange = [1000 5000 10000 25000 50000];
aneuFitRange = [-0.001 -0.005 -0.01 -0.05 -0.1];
aneuRateRange = [0.005 0.01 0.02 0.03 0.04];
expRange = [0.008 0.019796928 0.048989795 0.12123093 0.3];
nicheEffectRange = [0.7 0.75 0.8 0.85 0.9];
nicheEffectIncRange = [0.0005 0.001 0.005 0.025 0.05];
%>>>>>>>>>>>>>>>>>>>>
nConds = 5;
%>>>>>>>>>>>>>>>>>>>>

colorstring = 'mcrgbk';
legs = [];
legsLength = 145;

posTracker = 0;
% >>>>>>GLOBAL STORAGE>>>>>>>>>>>
globalAneu = [];
globalHSC = [];
globalDivStore = [];
% <<<<<<<<<<<<<<<<<<<<<<<<<<<<<<<<

for c = 1 : nConds

    cellDivRate = cellDivRateRange(3);
    initPopSize = initPopSizeRange(1);
    maxPopSize = maxPopSizeRange(3);
    aneuFit = aneuFitRange(5);
    aneuRate = aneuRateRange(2);
    expCoeff = expRange(c);
    nicheEffect = nicheEffectRange(1);
    nicheEffectInc = nicheEffectIncRange(3);
    %$$$$$$$$$$$$$$$$$$$$$$$$$$$$$$$$$$$$$4

    clearvars HSCpool
    timer = tic;

    % initial parameters:
    runs = 100; % number of simulation repeats
    maxPool = maxPopSize; % maximum capacity of the pool (max number of HSC)
    maxTime = 600; % maximum age (99 years)
    initHSC = initPopSize;
    %growthRate = 57;
    cellDivInit = 3;
    cellDivFin = 40;

    %===BEST==
    % aneuRate = 0.005;
    % aneuFit = -0.01;
    % nicheEffect = 0.83;
    % nicheEffectInc = 0.005;
    %=========

    % STORAGE MATRICES ------------------------------------
    HSCs = [];
    aneuStore = [];
    cellDivStore = [];
    % -----------------------------------------------------



    %POOL CAPACITY WITH AGE (%-barred code is an alternative for HSC pool size changing over lifetime)
    % growthCurve = [initHSC];
```

```
    % for i = 2 : maxTime
    %         %range = 0.3 0.12123093 0.048989795 0.019796928 0.008
    %         growthCurve(i) = growthCurve(i-1) + 0.5*growthRate*(1 - (growthCurve(i-1)/maxPool));
    % end

    oldCapacity = initHSC;
    growthCurve = [initHSC];
    for i = 1 : maxTime
        %range = 0.3 0.12123093 0.048989795 0.019796928 0.008
        newCapacity = oldCapacity + (round(expCoeff * oldCapacity * ((maxPool - oldCapacity) /
maxPool)));
        growthCurve = [growthCurve, newCapacity];
        oldCapacity = newCapacity;
    end

    for run = 1 : runs % this loop contains the whole run of the program for each individual

        disp(run)%------------------------------------------ for visualization only

        %//// INITIAL CELL POOL ////////////////////////////////////////////////////////////
        %-- 1. cell IDs
        %-- 2. cell ages
        %-- 3. fitness
        HSCpool = [];
        initAneu(1, 1:initHSC) = binornd(1, aneuRate, 1, initHSC); % aneuploidy trial
        %* plugging in initial aneuploid cells by IDS
        HSCpool(1, initAneu(1, 1:initHSC) == 0) = 1; % initial normal cells
        HSCpool(1, initAneu(1, 1:initHSC) == 1) = 2; % initial aneuploid cells
        %* cell ages
        HSCpool(2, 1:initHSC) = randi([2, cellDivInit], 1, initHSC); % initial cell ages
        %* normal and aneuploid cell fitness
        HSCpool(3, initAneu(1, 1:initHSC) == 0) = 1; % fitness of normal cells
        HSCpool(3, initAneu(1, 1:initHSC) == 1) = 1 + aneuFit; % fitness of aneuploid cells
        %\\\\\\\\\\\\\\\\\\\\\\\\\\\\\\\\\\\\\\\\\\\\\\\\\\\\\\\\\\\\\\\\\\\\\\\\\\\\\\\\\\\\

        nicheEffectLocal = nicheEffect;
        nicheEffectLocalInc = nicheEffectInc;

        for currentTime = 1 : maxTime

            %disp('current time')
            %disp(currentTime)

            %cellDiv = cellDivInit + ((cellDivFin-cellDivInit)*(abs(size(HSCpool, 2)-
initHSC)/abs(maxPool-initHSC))); %proportional to expansion
            cellDiv = cellDivRate;

            % data collection
            HSCs(run, currentTime) = size(HSCpool, 2);
            aneuStore(run, currentTime) = numel(HSCpool(1, HSCpool(1, :)==2))/size(HSCpool, 2)*100;
            cellDivStore(run, currentTime) = cellDiv;
            % ---------------



            % CELL DIVISIONS

            divMatrix = normrnd(cellDiv, cellDiv/8, 1, size(HSCpool, 2));

            HSCpool(2, HSCpool(2, :) >= divMatrix(1, :)) = 1; % resets the time past division for cells
that divide
            newCells = HSCpool(:, HSCpool(2, :) == 1); %new cells
            HSCpool(:, HSCpool(2, :) == 1) = 0;
            HSCpool = (HSCpool(:, HSCpool(1, :) > 0));

            %== Aneuploidy
            currAneu(1, 1: size(newCells, 2)) = binornd(1, aneuRate, 1, size(newCells, 2));
            newCells(1, currAneu(1, :) == 1) = 2;
            newCells(3, currAneu(1, :) == 1) = 1 + aneuFit;
            %==


            %NEW CELLS ADDED TO THE POOL
            HSCpool = [HSCpool, newCells, newCells];

            %CELLS COMPETE FOR SPACE
```

```
            %         overkill = size(HSCpool, 2)/growthCurve(currentTime);
            %         invs = 1./HSCpool(3, :);
            %         difprobs = invs/sum(invs);
            %         difprobs = difprobs-(mean(difprobs));
            %         difprobs = difprobs+(1-(1/overkill));
            %         difprobs(difprobs < 0) = 0;
            %         difprobs(difprobs > 1) = 1;
            %         differen = binornd(1, difprobs(1, :));
            %         HSCpool(:, differen(1, :) == 1) = 0;
            %         HSCpool = HSCpool(:, HSCpool(1, :) > 0);

            w_prob = find(HSCpool(1, :));
            n = size(w_prob,2);
            w_prob(2, :) = (growthCurve(currentTime) / n) * (n * HSCpool(3, w_prob(1, :)) /
sum(HSCpool(3, w_prob(1, :)))); %weighed probabilities
            w_prob(2, w_prob(2,:) >= 1) = 1;
            w_prob(3, :) = binornd(1, w_prob(2, :)); % all cells compete for free niches
            HSCpool(:, w_prob(1, w_prob(3, :) == 0)) = 0; % all lost cells zeroed
            w_prob = [];
            HSCpool = HSCpool(:, HSCpool(1, :) > 0);


            if(isnumeric(nicheEffect) & isnumeric(nicheEffectInc))
                %disp(nicheEffect);
                %NICHE EFFECT
                w_prob = find(HSCpool(1, :));
                n = size(w_prob,2);
                w_prob(2, :) = nicheEffectLocal;
                w_prob(2, w_prob(2,:) >= 1) = 1;
                w_prob(3, :) = binornd(1, w_prob(2, :)); % all cells compete for free niches
                HSCpool(:, w_prob(1, w_prob(3, :) == 0)) = 0; % all lost cells zeroed
                %w_prob = [];
                HSCpool = HSCpool(:, HSCpool(1, :) > 0);
                nicheEffectLocal = nicheEffectLocal + (1 - nicheEffectLocal)*(1-exp(-
nicheEffectLocalInc*currentTime));
                nicheEffectStore(run, currentTime) = nicheEffectLocal;
            end



            % CELL AGES UPDATED
            HSCpool(2, :) = HSCpool(2, :) + 1;
        end
        % MEMORY CLEANUP
        newCells = [];

        %     time = toc(timer);
        %     hours = floor(time / 3600);
        %     time = time - hours * 3600;
        %     mins = floor(time / 60);
        %     secs = time - mins * 60;
        %     secs = round(secs);
        %
        %     fprintf('Execution time (HH:MM:SS) - %d:%d:%d        \n\n', hours, mins, secs);


    end

    % writing into global storage
    globalAneu = [globalAneu; aneuStore];
    globalDivStore = [globalDivStore; cellDivStore];
    % ===========================


    for i = 1 : size(aneuStore, 2)
        averAneuStore(i) = mean(aneuStore(:, i));
    end

    if(isnumeric(nicheEffect) & isnumeric(nicheEffectInc))
        nicheEffectLocal = nicheEffect;
    end

    plot([1:maxTime], averAneuStore, 'Color', colorstring(c))
    %plot([1:maxTime], HSCs, 'Color', colorstring(c))
    hold on
    varToStr = @(x) inputname(1);
```

```
    leg = strcat(varToStr(aneuFit), '=', num2str(aneuFit), ':::', ...
        varToStr(aneuRate), '=', num2str(aneuRate), ':::', ...
        varToStr(expCoeff), '=', num2str(expCoeff), ':::', ...
        varToStr(nicheEffect), '=', num2str(nicheEffectLocal), ':::', ...
        varToStr(nicheEffectInc), '=', num2str(nicheEffectLocalInc), ':::', ...
        varToStr(initHSC), '=', num2str(initHSC), ':::', ...
        varToStr(maxPool), '=', num2str(maxPool), ':::', ...
        varToStr(cellDivRate), '=', num2str(cellDivRate), ':::' ...
        );
    remn(1:legsLength-size(leg, 2)) = '<';

    legs = [legs; [leg, remn]];
    remn=[];

    time = toc(timer);
    hours = floor(time / 3600);
    time = time - hours * 3600;
    mins = floor(time / 60);
    secs = time - mins * 60;
    secs = round(secs);

    fprintf('Execution time (HH:MM:SS) - %d:%d:%d        \n\n', hours, mins, secs);

end

legend('show')
```

Figures

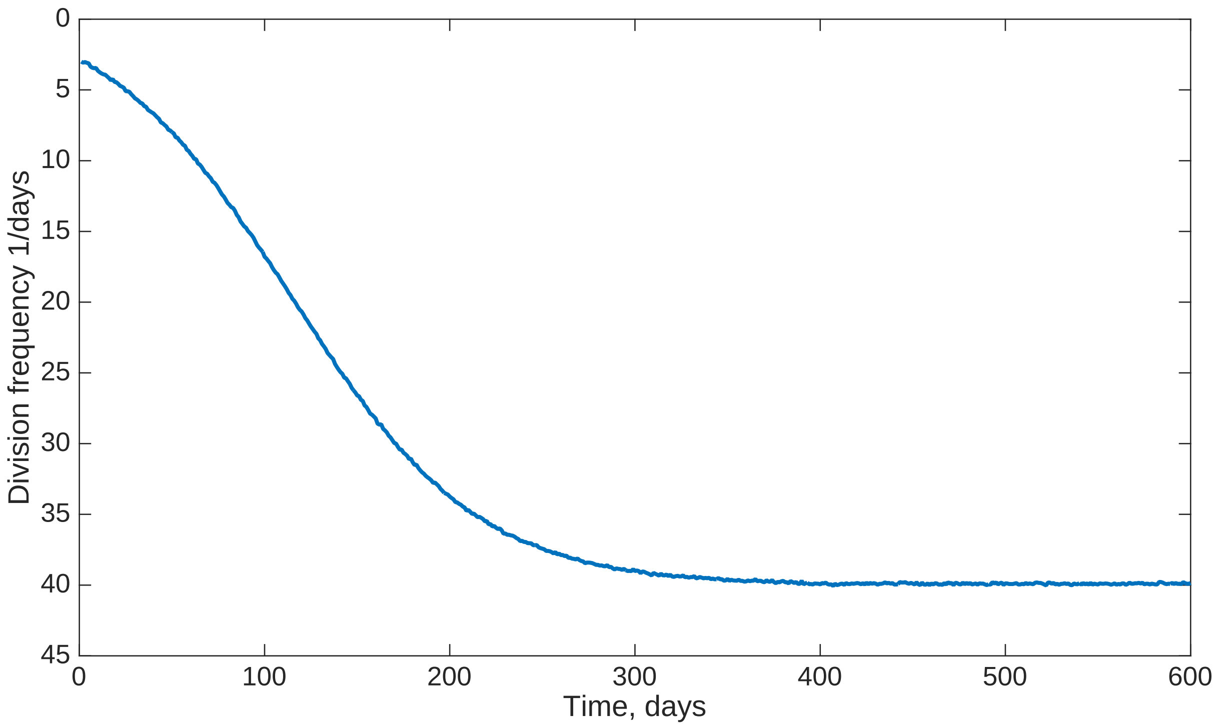

Fig. S1. Dynamics of changing average cell division rate over time (typical example)

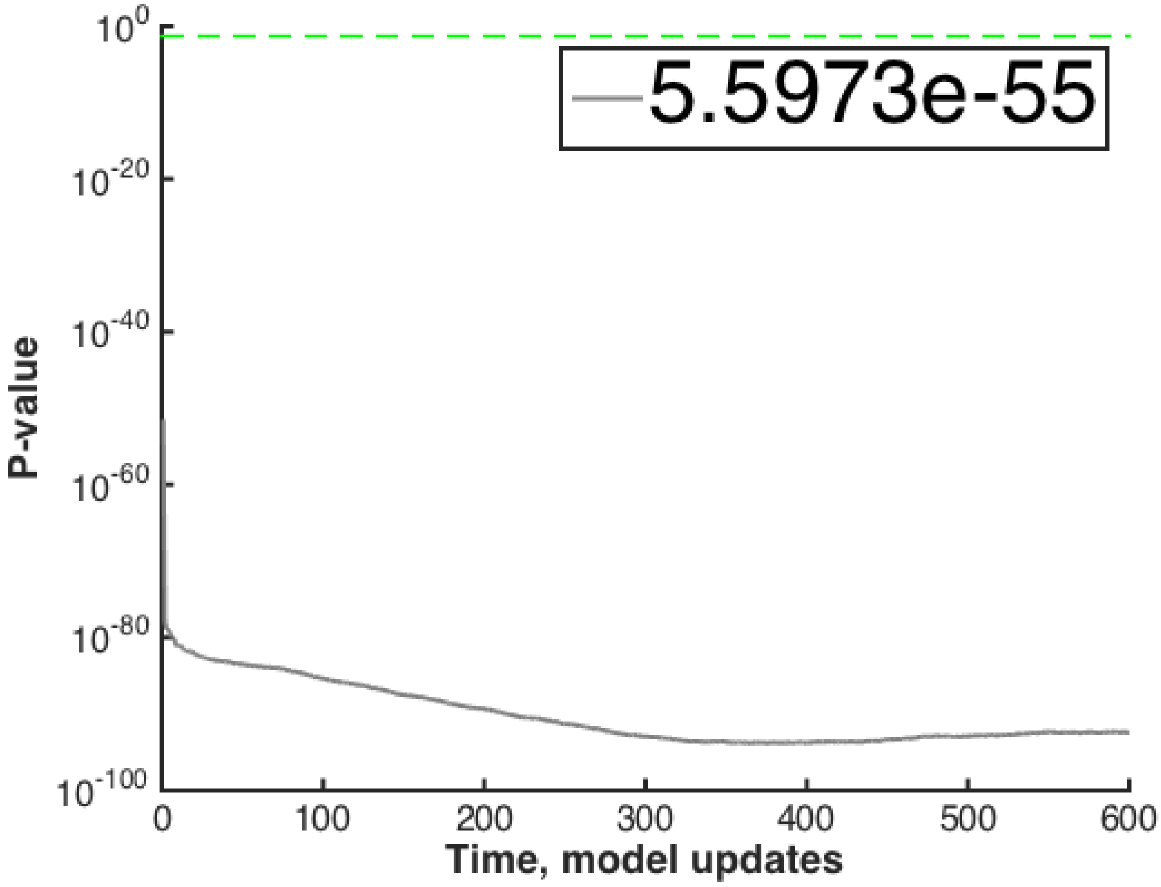

Fig. S2. Kruskal-Wallis analysis of the differences between the processes shown Fig. 1B. At each simulation time point, a p-value (Y-axis) was calculated by comparing the modeled processes each represented by a sample of 100 repeated runs using the Kruskal-Wallis method

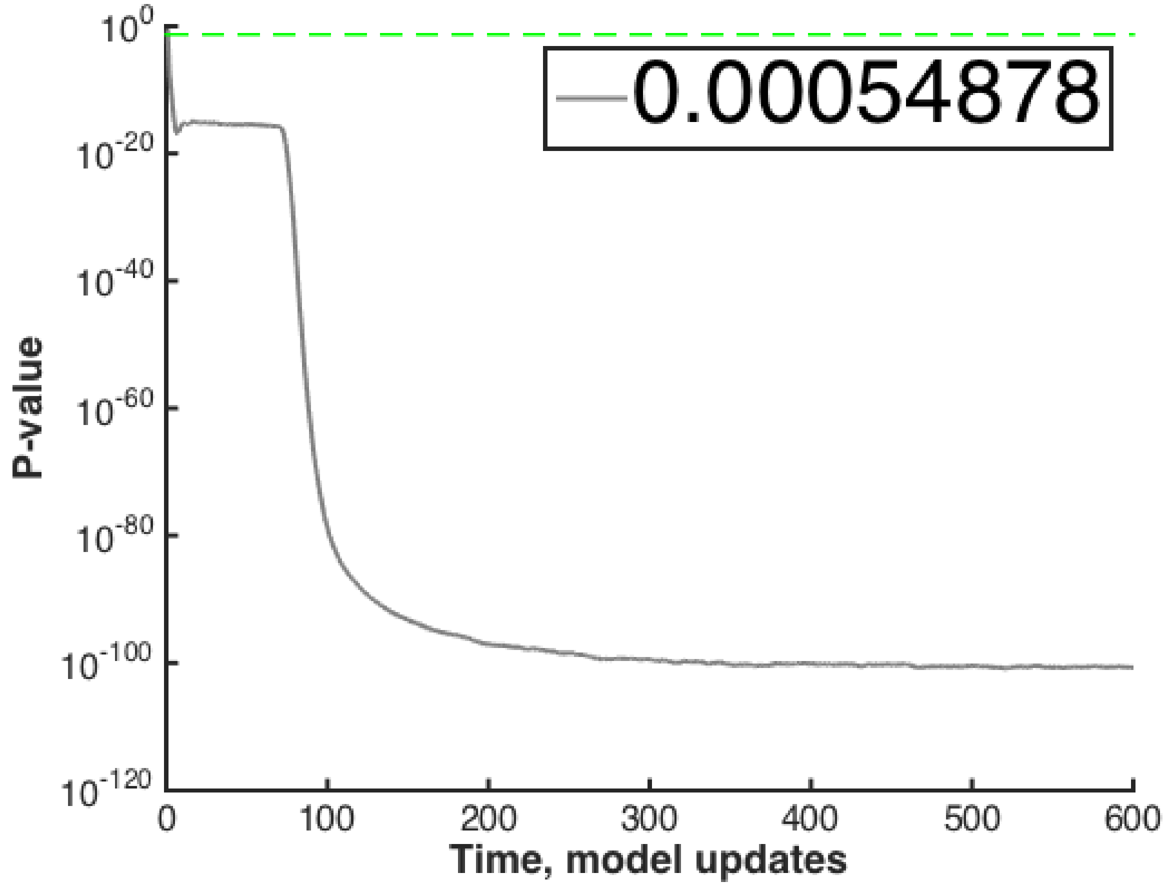

Fig. S3. Kruskal-Wallis analysis of the differences between the processes shown Fig. 1C. At each simulation time point, a p-value (Y-axis) was calculated by comparing the modeled processes each represented by a sample of 100 repeated runs using the Kruskal-Wallis method

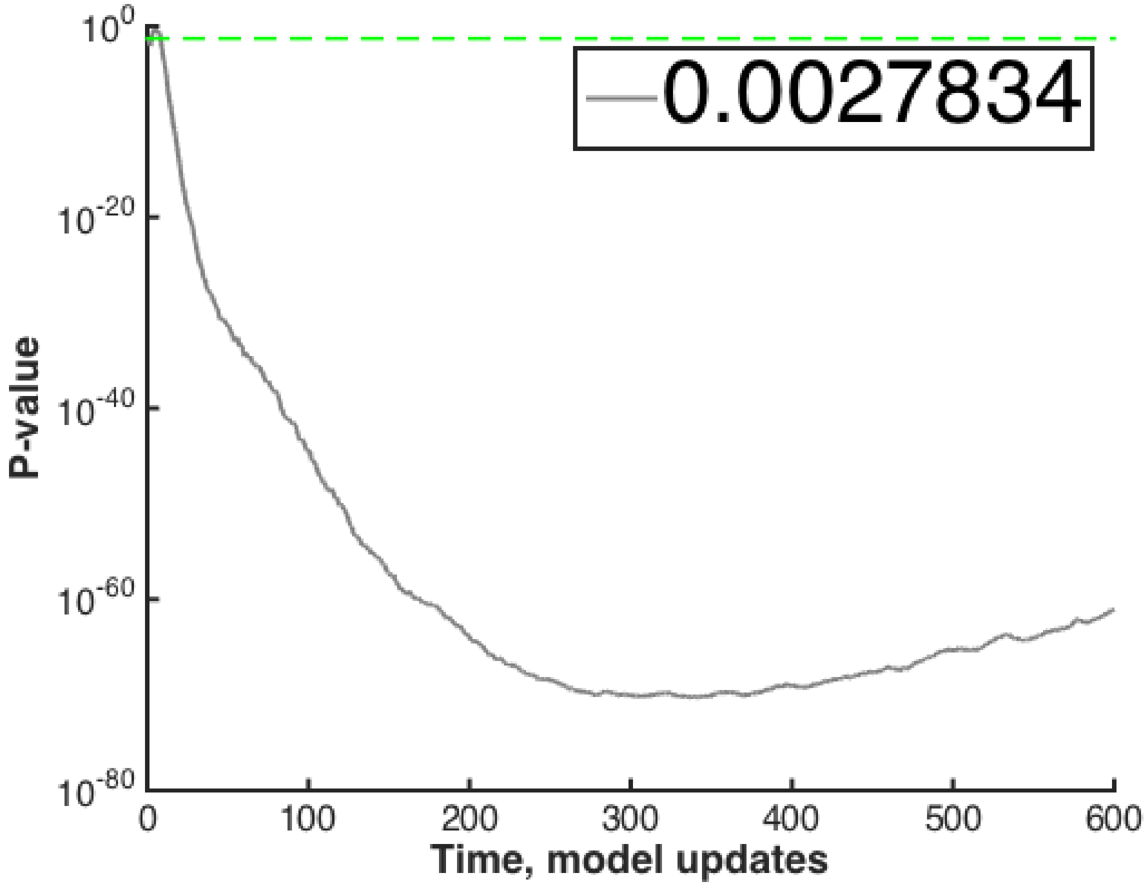

Fig. S4. Kruskal-Wallis analysis of the differences between the processes shown Fig. 1E. At each simulation time point, a p-value (Y-axis) was calculated by comparing the modeled processes each represented by a sample of 100 repeated runs using the Kruskal-Wallis method

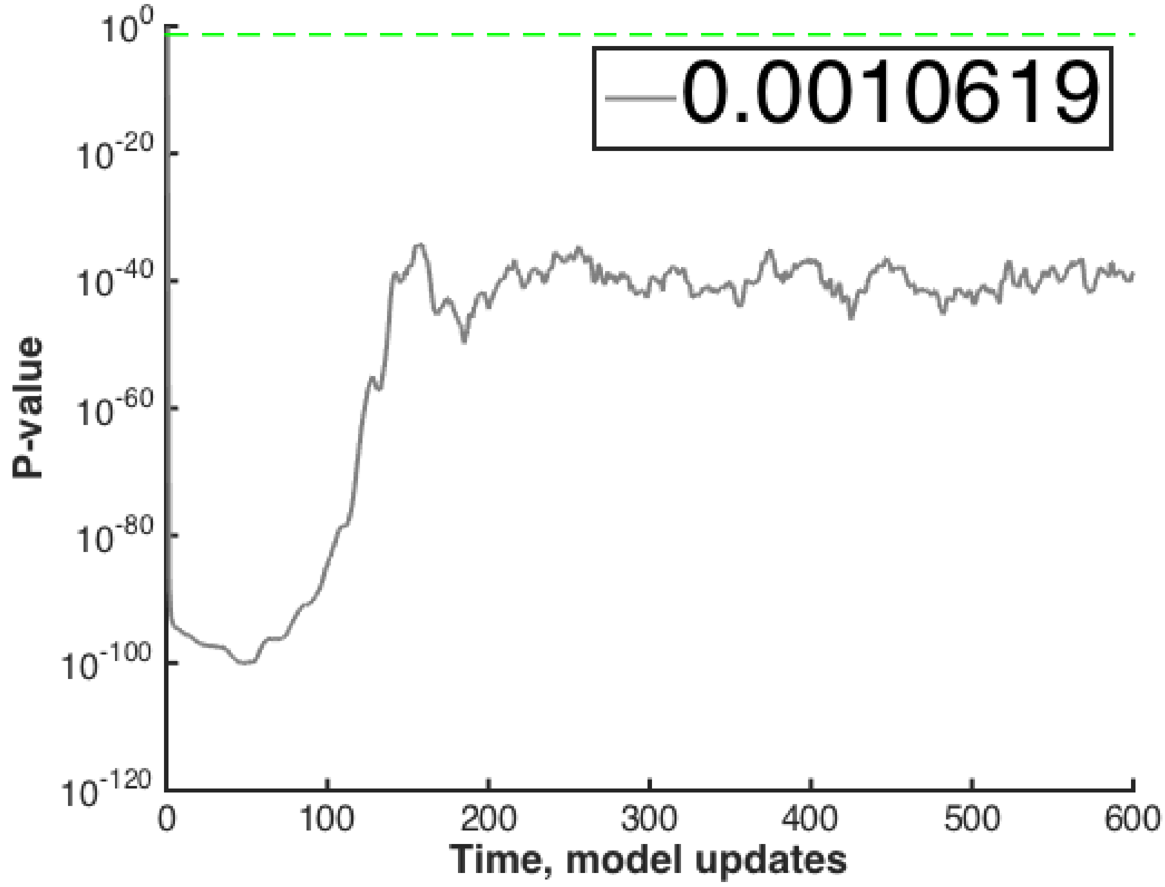

Fig. S5. Kruskal-Wallis analysis of the differences between the processes shown Fig. 1F. At each simulation time point, a p-value (Y-axis) was calculated by comparing the modeled processes each represented by a sample of 100 repeated runs using the Kruskal-Wallis method

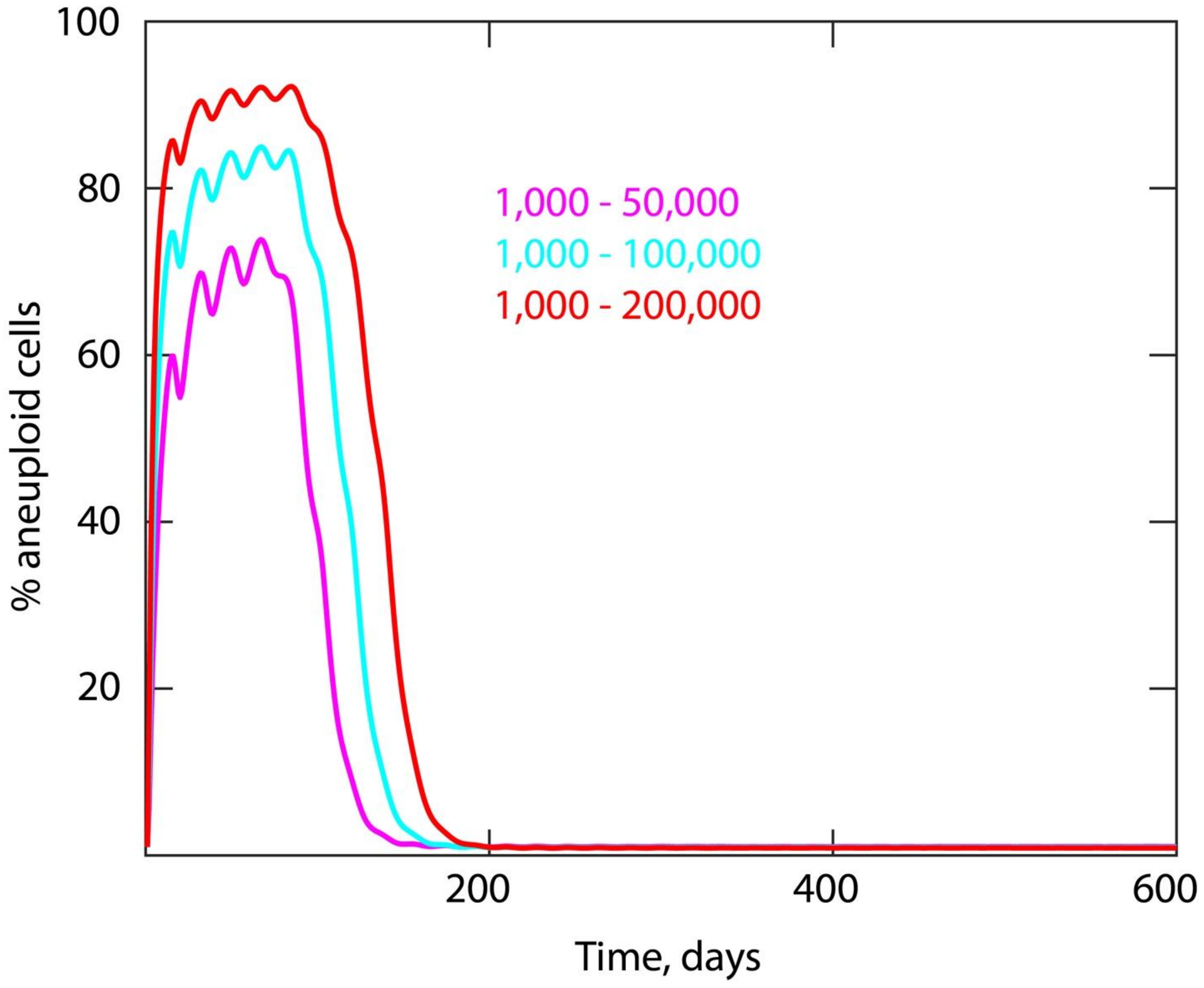

Fig. S6. Dynamics of aneuploid cells at different final cell pool sizes. Color matched labels indicate the initial and final cell pool sizes and are color matched to the corresponding aneuploid cell dynamics curves. Parameters other than the final pool size are as in Fig. 1F.

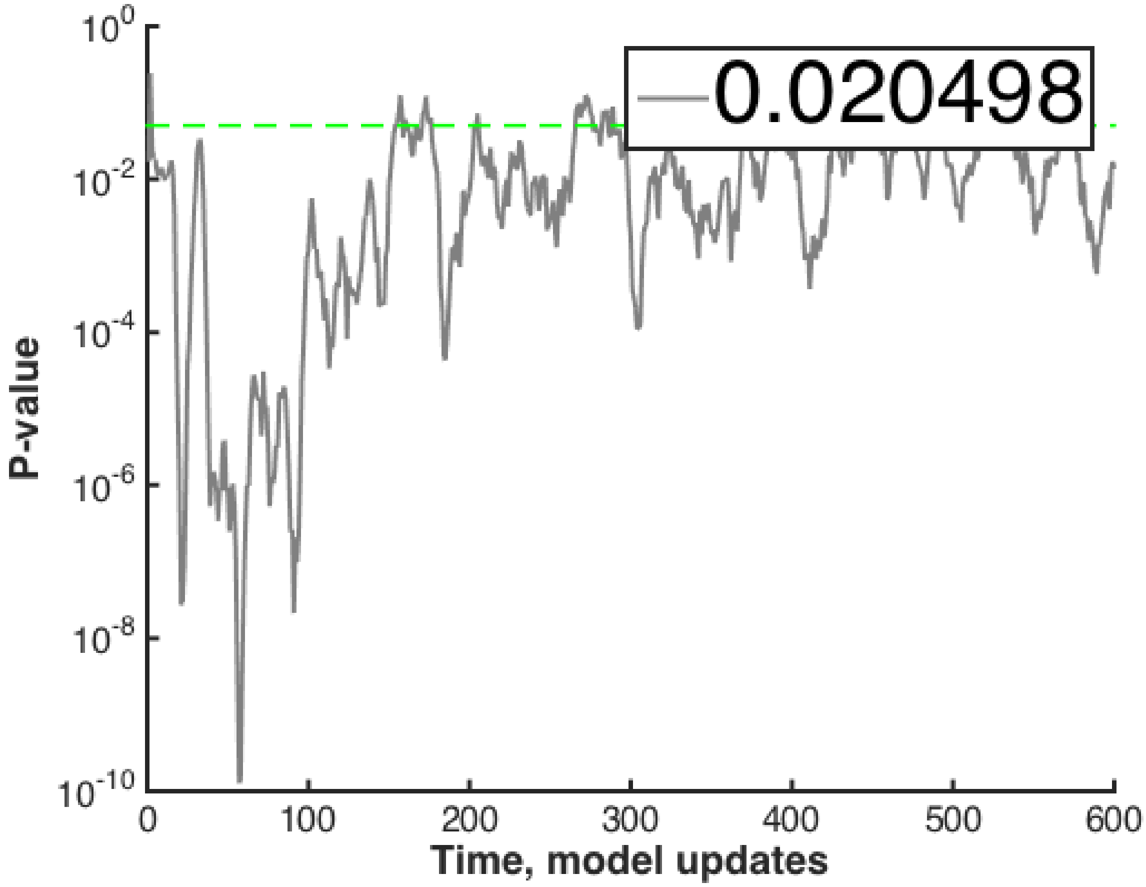

Fig. S7. Kruskal-Wallis analysis of the differences between the processes shown Fig. 1G. At each simulation time point, a p-value (Y-axis) was calculated by comparing the modeled processes each represented by a sample of 100 repeated runs using the Kruskal-Wallis method

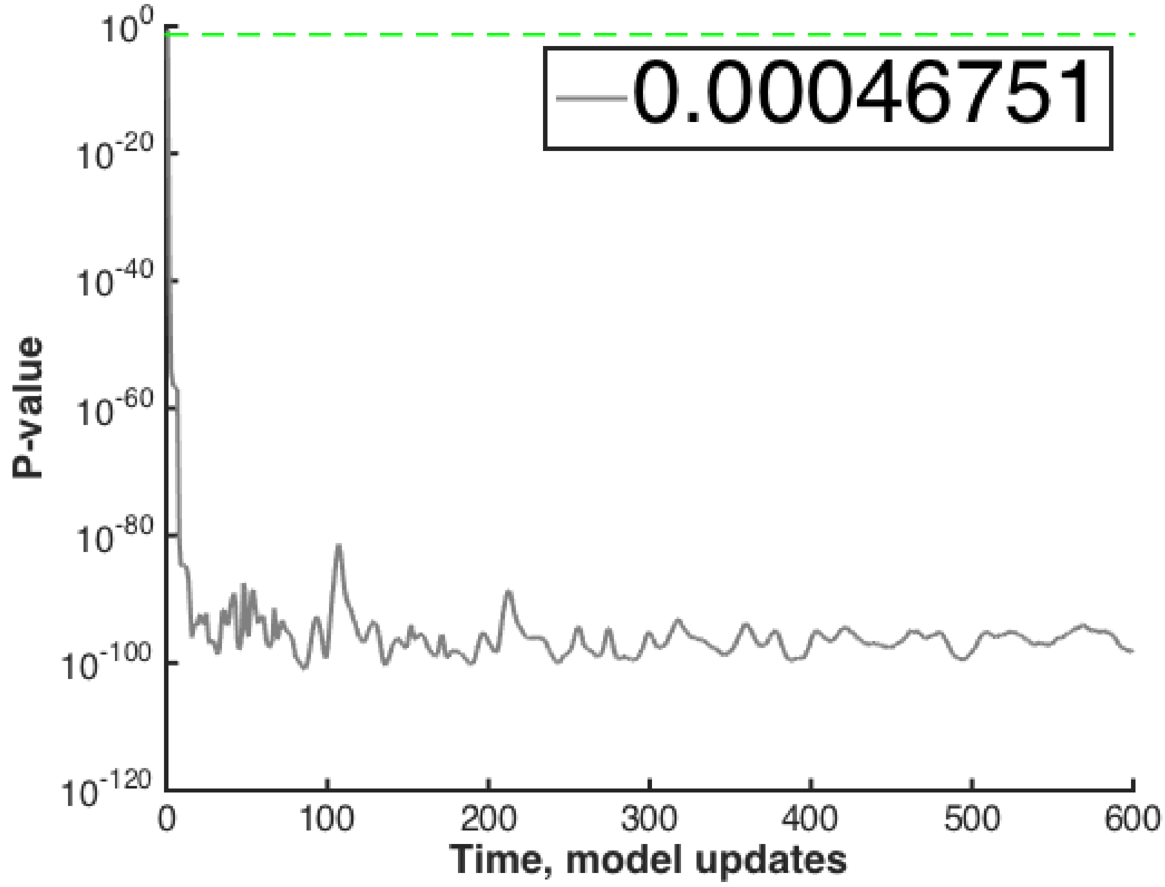

Fig. S8. Kruskal-Wallis analysis of the differences between the processes shown Fig. 1H. At each simulation time point, a p-value (Y-axis) was calculated by comparing the modeled processes each represented by a sample of 100 repeated runs using the Kruskal-Wallis method

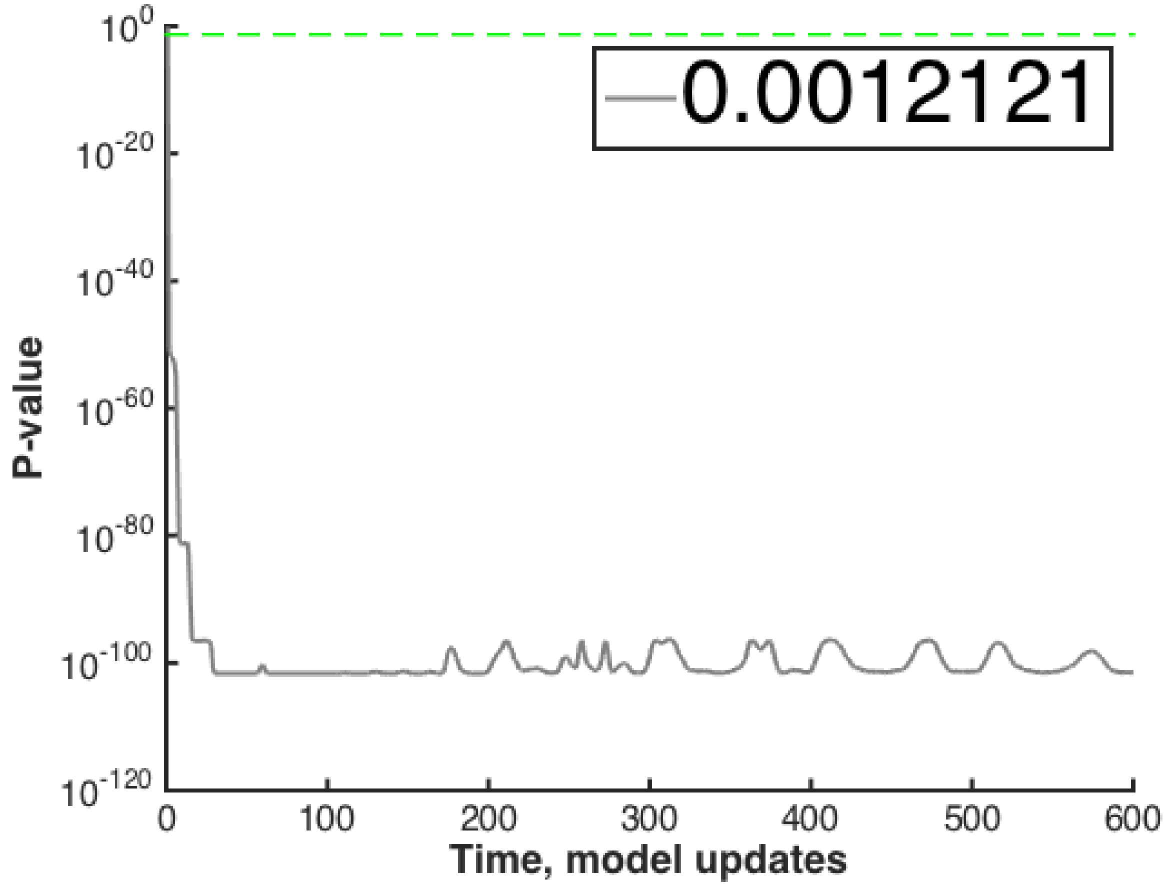

Fig. S9. Kruskal-Wallis analysis of the differences between the processes shown Fig. 1I. At each simulation time point, a p-value (Y-axis) was calculated by comparing the modeled processes each represented by a sample of 100 repeated runs using the Kruskal-Wallis method

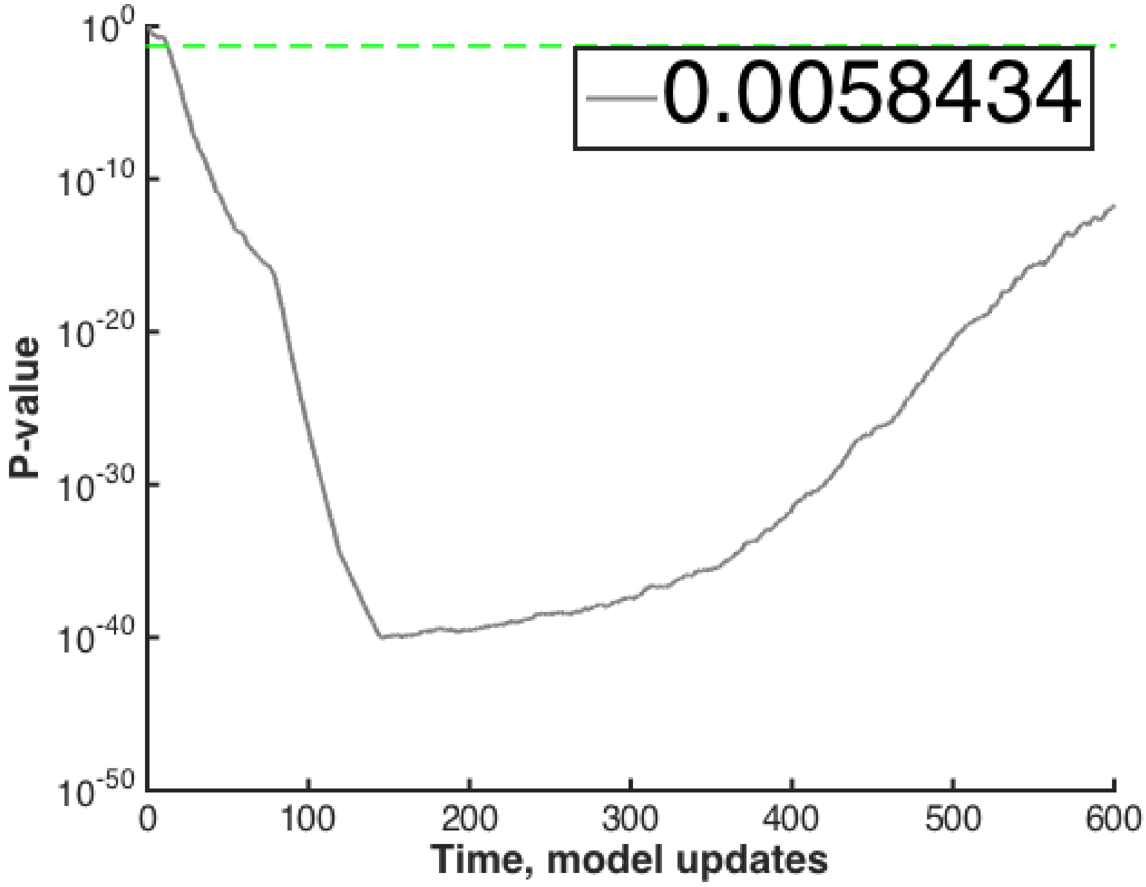

Fig. S10. Kruskal-Wallis analysis of the differences between the processes shown Fig. 2C. At each simulation time point, a p-value (Y-axis) was calculated by comparing the modeled processes each represented by a sample of 100 repeated runs using the Kruskal-Wallis method

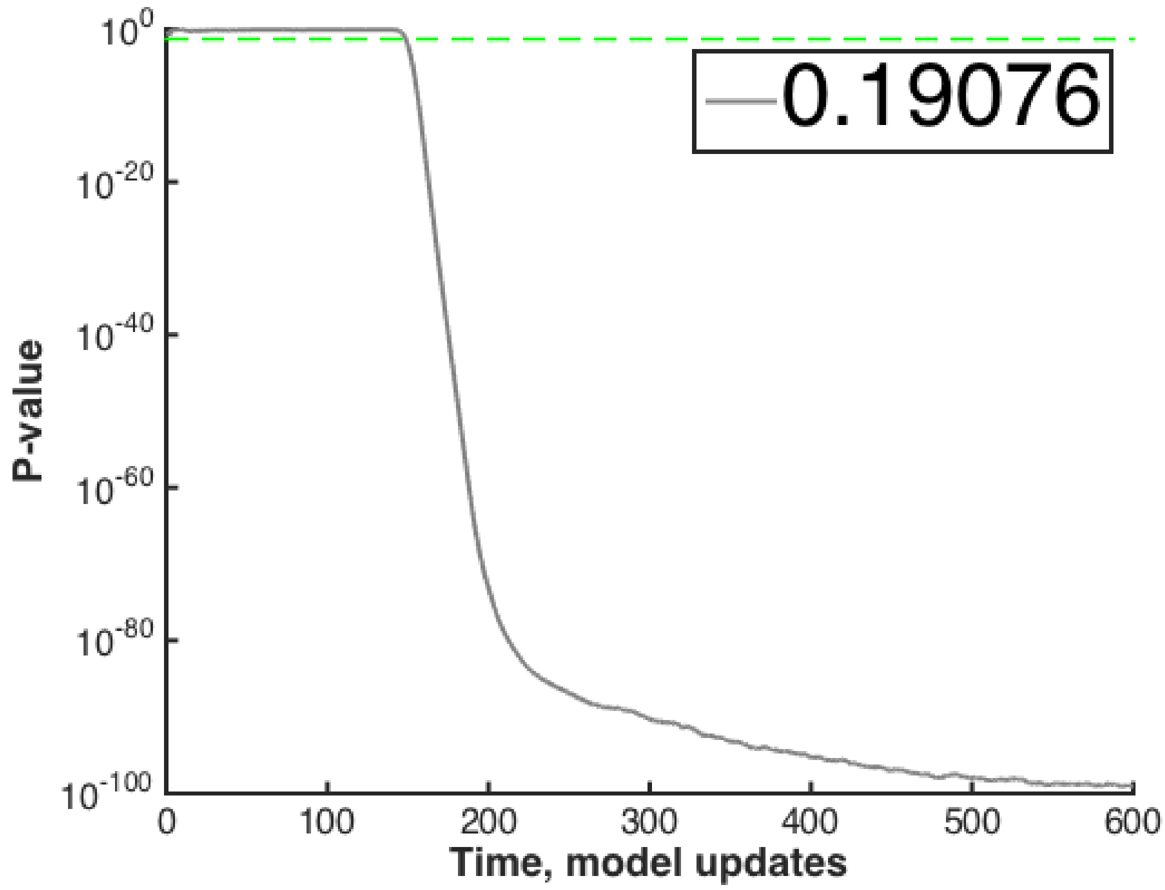

Fig. S11. Kruskal-Wallis analysis of the differences between the processes shown Fig. 2D. At each simulation time point, a p-value (Y-axis) was calculated by comparing the modeled processes each represented by a sample of 100 repeated runs using the Kruskal-Wallis method

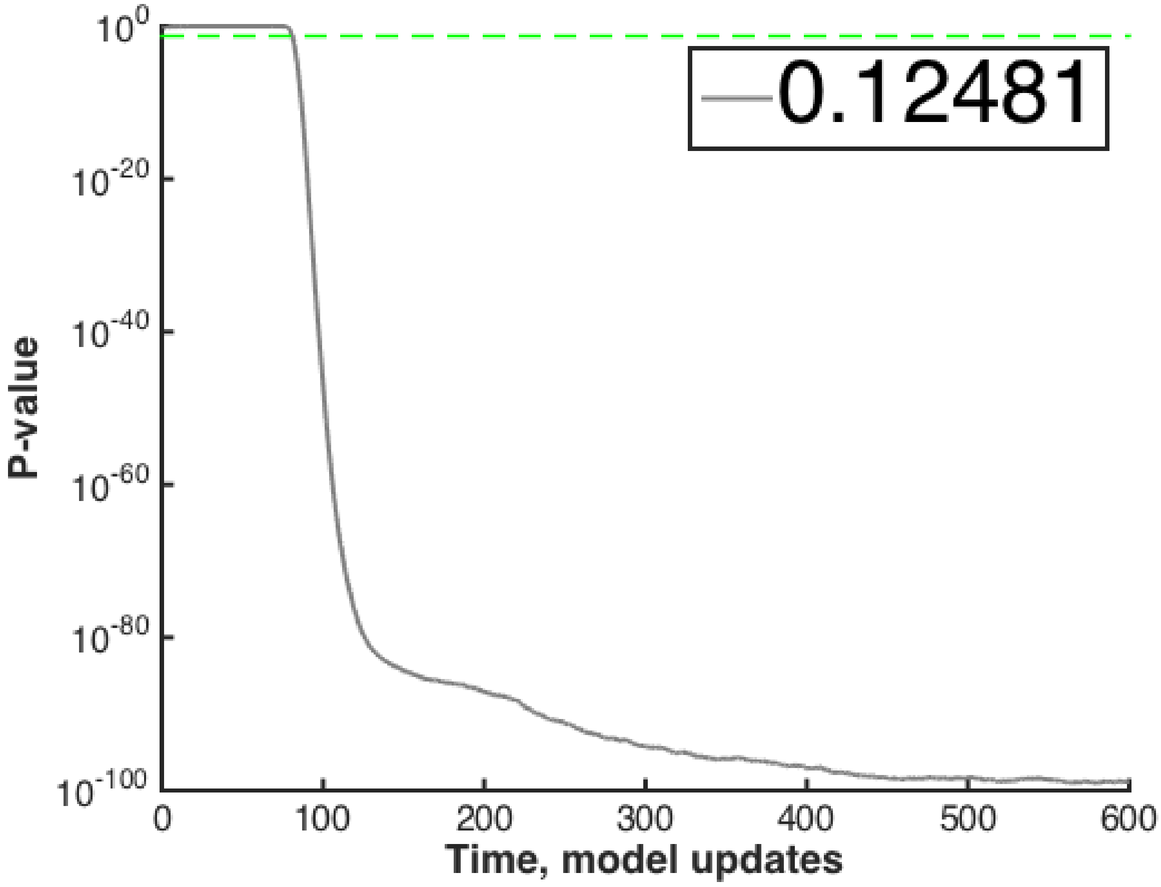

Fig. S12. Kruskal-Wallis analysis of the differences between the processes shown Fig. 2E. At each simulation time point, a p-value (Y-axis) was calculated by comparing the modeled processes each represented by a sample of 100 repeated runs using the Kruskal-Wallis method

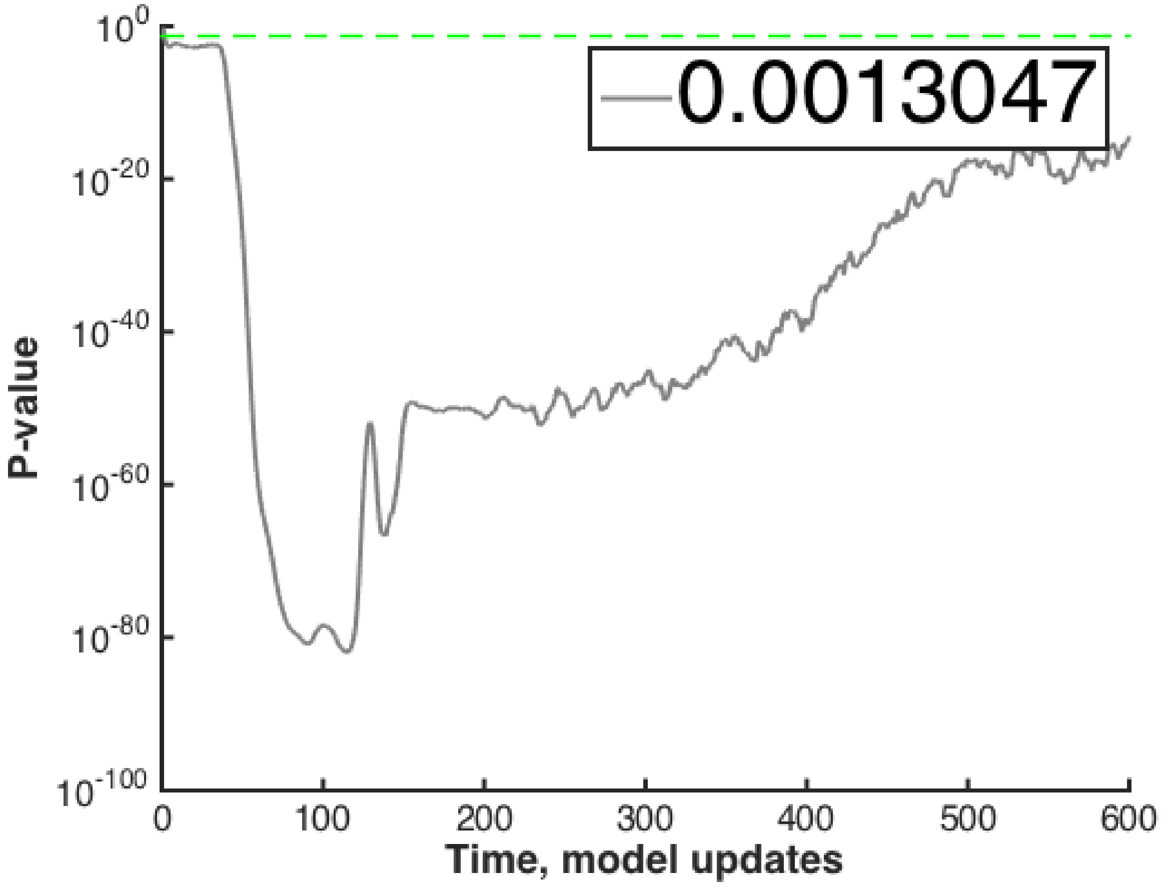

Fig. S13. Kruskal-Wallis analysis of the differences between the processes shown Fig. 3A. At each simulation time point, a p-value (Y-axis) was calculated by comparing the modeled processes each represented by a sample of 100 repeated runs using the Kruskal-Wallis method

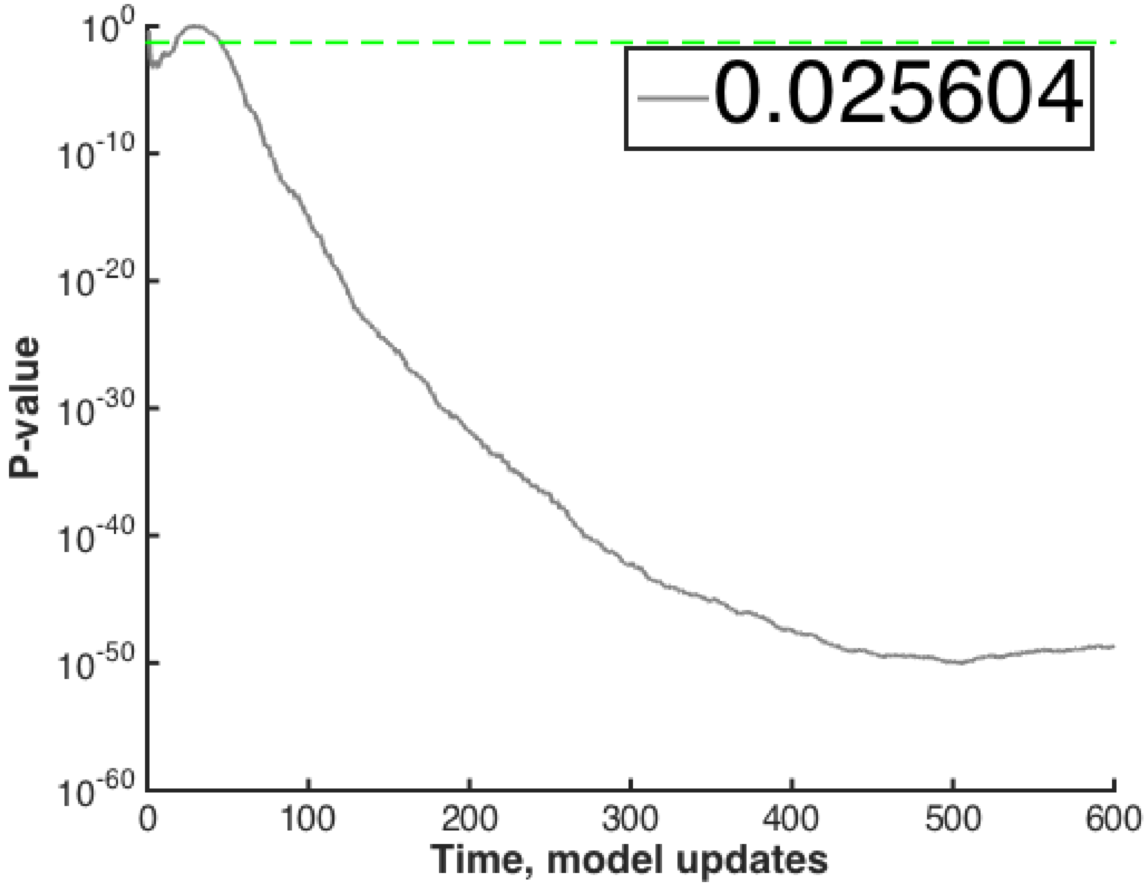

Fig. S14. Kruskal-Wallis analysis of the differences between the processes shown Fig. 3B. At each simulation time point, a p-value (Y-axis) was calculated by comparing the modeled processes each represented by a sample of 100 repeated runs using the Kruskal-Wallis method

Aneuploidy counts

Sample	# Aneuploid	# Euploid	Total Cells	Percent Aneuploid	Number of Animals	Days Post Recons-titution	Published in Pfau *et al.* 2016?
3 weeks FL	1	11	12	8.3	2	21	yes
3 weeks BM	1	17	18	5.6	2	21	yes
6 weeks FL	6	19	25	24.0	3	42	yes
6 weeks BM	6	19	25	24.0	3	42	yes
13 weeks FL	4	12	16	25.0	2	91	yes
13 weeks BM	3	13	16	18.8	2	91	yes
36 weeks FL	1	11	12	8.3	2	252	yes
34 weeks BM	2	10	12	16.7	2	238	yes
52 weeks FL	0	12	12	0.0	1	364	no
50 weeks BM	0	11	11	0.0	2	350	no
